# Circulating MicroRNAs As Potential Biomarkers for Veterinary Infectious Diseases

**DOI:** 10.3389/fvets.2017.00186

**Published:** 2017-11-01

**Authors:** Hao Dong, Qiang Gao, Xiaowei Peng, Yu Sun, Tao Han, Bolin Zhao, Yufu Liu, Chuanbin Wang, Xiaohui Song, Jiajun Wu, Lin Yang

**Affiliations:** ^1^National Veterinarian Diagnostic Center, China Animal Disease Control Center, Beijing, China; ^2^Department of Inspection Technology Research, China Institute of Veterinary Drug Control, Beijing, China; ^3^South China Agricultural University, Guangzhou, China

**Keywords:** circulating microRNA, biomarkers, infectious diseases, veterinary, diagnosis

## Abstract

MicroRNAs (miRNAs) are a kind of small non-coding RNA molecules that could regulate multiple biological pathways at posttranscriptional level. Over 2,000 miRNAs have so far been discovered in humans, and many of them are found to be linked to various kinds of diseases. Thus, miRNAs are being considered as clinical diagnostic and therapeutic targets. With the discovery of high stability of circulating miRNAs in various kinds of mammalian body fluids, the potential of circulating miRNAs as diagnostic/prognostic biomarkers of infectious diseases aroused great interest among researchers. As far as human diseases are concerned, some biomarkers based on circulating miRNAs have been progressed to clinical application. In veterinary fields, however, this concept is only beginning to come into view. In this review, we summarize an update of preclinical studies on using circulating miRNAs as diagnostic biomarkers to combat infectious diseases that affect domestic animals.

## Introduction

MicroRNAs (miRNAs) are small, endogenously expressed, non-coding RNA transcripts, about 22 nucleotides in length, with unique sequences targeting mRNAs for posttranscriptional regulation. Various researches have showed that miRNAs play important roles in various physiological and pathological processes, such as immune response, inflammatory response, and tumor occurrence (1, 2). The dysregulations of miRNAs have also been implicated in various kind of diseases, including diabetes, kidney disease, cancer, and many infectious diseases. Since 2008, the stable existence of miRNAs was identified in different type of body fluids (such as urine, serum, and plasma) (3–5). The application prospect of circulating miRNAs as non-invasive diagnostic biomarkers of disease status attracts the interest of researchers (5–7). At present, compared to the studies of circulating miRNAs in human diseases, only very limited studies are focused on miRNAs in veterinary infectious diseases and most are related to cellular miRNAs, instead of circulating miRNAs. Recently, the significance of functions of miRNAs in the host response to several veterinary virus diseases has been comprehensively reviewed (8, 9). In this review, we mainly focus on the research progress of using circulating miRNAs as diagnostic biomarkers for infectious diseases of domestic animals.

## Function of miRNAs

Over 30 years ago, the first miRNA was identified in the nematode *Caenorhabditis elegans* with the identification of the developmental regulator *lin-4* (10). In the year of 2000, another miRNA, let-7, regulating the timing of *C. elegans* development, was found to be conserved in a wide range of animal species, including human, and this result indicates this kind of small regulatory RNAs has a more general biological function (11, 12). In 2002, Calin and colleagues found that miR-15 and miR-16 were tumor suppressors for chronic lymphocyte leukemia, which demonstrated for the first time the relationship between dysregulation of intracellular miRNAs and disease (13). Shortly afterward, researchers found that the expression levels of let-7 were closely related to prognosis for lung cancer survival (14). More and more research literatures have highlighted that alterations in miRNA expression profile were related to a wide range of human diseases, especially in the studies of various cancers (15–17).

In terms of biological function of miRNAs, it was first reported in the researches about mouse models deficient for Dicer and DGCR8. DGCR8 plays an important role in miRNA biogenesis, while Drosha participates in other RNA metabolic pathways. Therefore, knockout of the two genes resulted in loss of most miRNAs and embryonic lethality (18, 19). However, individual miRNAs are seems to be not required for specification of individual tissues. In addition, miRNAs are usually required for maintaining tissue homeostasis, since the expression levels of many tissue-restricted miRNAs are usually downregulated in the process of illness (12).

Currently, over 2,000 miRNAs have been reported according to results of human transcriptome, and it is believed that over 60% of coding genes are regulated by miRNAs in the genome (20). These findings indicate miRNAs may participate in the vast majority of physiological pathways. Recently, the immunoregulatory roles of miRNAs during infection have been widely studied. For example, various miRNAs have been demonstrated to regulate the toll-like receptor 4 pathway in the host innate immune response (21, 22).

## Circulating miRNAs as Diagnostic Biomarkers

A large number of studies have demonstrated that dysregulation of circulating miRNAs are associated with a wide variety of diseases progression and syndromes. At present, the relationships between profile changes of circulating miRNAs and human diseases (such as virus infections, cancers, and liver injury) have been widely reported (5, 23, 24). In the respect of veterinary infectious diseases, many studies have also demonstrated the correlation between expression profiles of circulating miRNAs and various bacteriosis (Paratuberculosis), virus disease [foot-and-mouse disease, bovine viral diarrhea (BVD)], and parasitosis (Echinococcosis).

Immune and non-immune cells could actively release miRNAs into extracellular environments (25, 26). In 2008, researchers found for the first time that circulating miRNAs were present in the samples of serum and plasma. Subsequently, circulating miRNAs were also found in other body fluids, such as saliva, urine as well as semen (27, 28). For a long time period, invasive tissue biopsies served as the main diagnostic methods for cancer; however, the existence of circulating miRNAs in blood samples of cancer parents undoubtedly significantly promotes the development of diagnostic techniques for cancer (4, 5, 29, 30). For some infectious diseases that lack of blood-based diagnostic methods, circulating miRNA is likely a good choice in term of establishing new diagnostic methods. For instance, human tuberculosis is an infectious disease that a number of researches have established new diagnostics methods using circulating miRNAs, and currently, dozens of circulating miRNAs have been identified related to this disease. However, the results obtained by different researchers exhibited somewhat inconsistency, which may be due to the different test samples (serum and sputum), different test technologies (miRNA-seq, microarray, and RT-qPCR), and data normalization methods (31).

In addition, circulating miRNAs are remarkably stable under harsh conditions, such as extended storage, boiling, low or high pH, as well as multiple freeze–thaw cycles (3, 5, 23). A study of detecting bovine serum samples from experimental infections with *Mycobacterium avium* subspecies paratuberculosis (MAP) demonstrated that the circulating miRNA profile of samples, which were stored at −20°C for 10–15 years, was remarkably similar to that of fresh serum samples (stored at −80°C for less than one year) (7). In another study, researchers evaluated the stability of miRNA levels in plasma and serum from healthy dogs after storage at room temperature for different time points, and it was found that miRNAs were sufficiently stable in serum or plasma stored at room temperature for 1 h but not for 24 h (32). The remarkable stability of circulating miRNAs in body fluids is considered to be mainly attributed to two mechanisms: (1) circulating miRNAs could form a protein–miRNA complex with argonaute proteins or high-density lipo-proteins and (2) they could incorporate into exosomes (33).

Based on all these features of circulating miRNAs mentioned above, they have attracted great attention of researchers as a kind of potential diagnostic biomarker for various diseases.

## Analysis of Circulating miRNA

As mentioned previously, circulating miRNAs can be detected in many kind of biofluids, while majority of current studies about circulating miRNAs are based on serum and plasma. In addition, the abundance of circulating miRNAs varies in different type of body fluids (27, 34). Due to this reason, here we only focus on discussion of the main points of detecting circulating miRNAs in plasma or serum samples.

Previous studies about analysis of serum and plasma samples demonstrate that different sample treatment methods can affect the quality of extracted miRNAs (34, 35). For example, as heparin could inhibit downstream PCR assay, collection of peripheral blood samples should use EDTA-coated tubes (36, 37). In addition, while separation of serum or plasma, hemolysis should be strictly avoided, as intracellular miRNAs from platelets and erythrocytes can introduce significant bias in the expression profiles of circulating miRNAs (34, 35, 38). To determine whether the serum and plasma samples are contaminated by hemolysis, the relative expression levels of the specific miR-451 of erythrocyte and the stable miR-23a could be used as an indicator (35).

Currently, several miRNA extraction kits are available to extract circulating miRNAs from blood. In a recent study, Guo and colleagues compared the performance of five kits (ThermoFisher Scientific Ambion TRIzol LS Reagent, Qiagen Circulating Nucleic Acid Kit, QiaSymphony RNA extraction kit, Qiagen miRNEasy, and the Exiqon MiRCURY RNA Isolation Kit) for isolating circulating extracellular sRNAs, including miRNAs, and their variant isoforms (isomiRs), and transfer RNA-derived small RNAs (tDRs), and other miscellaneous sRNAs. In the respect of isolation of miRNAs, Ambion TRIzol LS produced the most reads for miRNAs, the repeatability of QiaSymphony RNA extraction kit was the highest, and Circulating Nucleic Acid Kit detected the greatest number of miRNAs. In addition, Circulating Nucleic Acid Kit also detected the most singleton miRNAs, which could explain why this kit was able to detect the most miRNAs (39).

As miRNAs are different from mRNAs in the respects of biochemistry and molecular structure, the techniques for miRNAs detection are not completely the same as that of mRNAs detection (40–42). Here, we mainly describe three frequently used techniques of miRNAs detection.

Reverse transcription quantitative real-time PCR is the most widely used technology for detecting miRNA expression level. As the sequences of miRNAs are very short, the experimental flow is different from the conventional reverse transcription PCR of mRNAs. At present, there are two common strategies used to detect the expression of circulating miRNAs: (1) using stem-loop reverse transcription followed by TaqMan PCR analysis (43) and (2) polyadenylated and reverse transcribed by a poly(T) adapter for quantitative RT-PCR using the miRNA-specific forward primer and sequence complementary to the poly(T) adapter as the reverse primer (44). It is worth noting that RT-qPCR-based technologies are unable to identify new miRNAs and special attention is needed for the design of standardized internal controls (40, 45, 46).

Hybridization-based technologies usually depend on DNA capture probes that are immobilized on a microarray so that the fluorescent signal intensities could be quantified to evaluate expression levels of different miRNAs. At present, many commercial miRNA array products are available, such as GeneChip^®^ miRNA Arrays (Affymetrix), miRCURY LNA™ microRNA Arrays (Exiqon), and SurePrint miRNA Microarrays (Agilent) (47). Compared to other methods, the specificity and dynamic range of hybridization-based technologies are relatively lower. Thus, the results of microarray methods are usually needed to verify by RT-qPCR (48). Recently, the NanoString Technologies, Inc. established a new hybridization-based method (nCounter^®^ miRNA Expression Assay) that does not require a PCR amplification step or direct labeling of target miRNAs (49). This approach has several advantages: it is as sensitive as RT-PCR; it has high throughput; and it could test up to 800 distinct miRNA variant targets in the same assay (31). However, similar to RT-qPCR, these hybridization-based methods are unable to identify novel miRNAs.

Unlike the methods mentioned above, miRNA-seq methods are able to discover global expression profiling of the whole miRNA transcriptome from certain biological sample (50). Compared to microarrays, the most considerable advantage of miRNA-seq is that it does not limit to detection of known miRNAs. miRNA-seq starts with constructing a cDNA sequence library reversely transcribed from short sRNAs selected by different methods, for example, size-selected gel electrophoresis. The prepared, indexed, and pooled cDNA library is subsequently sequenced using different sequencing platforms. However, special attention should be paid that technical biases inherent to different sequencing technologies may generate unreal reads (51, 52).

## Differential Expression of Circulating miRNAs in Veterinary Infectious Diseases

### Foot-and-Mouth Disease (FMD)

Foot-and-mouth disease, caused by foot-and-mouth disease virus, is a highly contagious infectious disease with significant economic impact. FMD can affect livestock and wild cloven-hoofed animals worldwide. This disease is characterized by fever and blister-like sores in the mouth, on the teats, on the tongue and lips, and between the hooves.

Using miRNA PCR array plates, Stenfeldt and colleagues detected the alterations of miRNA levels in bovine serum samples collected from three different phases of FMDV infection (acute, persistent, and convalescent phases) compared to uninfected animals. A total of 169 abundant miRNAs were detected in serum samples, and 3 differentially expressed miRNAs were identified: the expression of miR-1281 was significantly reduced at both acute and persistent infection stages; bta-miR-17-5p was expressed in the highest level at acute infection stage, whereas the expression level of bta-miR-31 was the highest during FMDV persistence. In addition, as cattle that cleared infection resembled the baseline profile, the authors considered that serum miRNA profiling could be used for identification of subclinically infected FMDV carriers (53).

### Bovine Viral Diarrhea

Bovine viral diarrhea virus (BVDV) is an important pathogen of cattle with a global distribution and causes major economic losses (54). Animals infected with BVDV could exhibit a variety of clinical symptoms including diarrhea, depression, and pyrexia ranging from clinically mild to severe. However, in many cases, the symptoms of BVDV infections are subclinical and difficult to detect (55–57).

To identify circulating miRNAs that could be used for biomarkers indicating the timing of BVDV exposure to cattle, Taxis and colleagues investigated miRNA profiles of BVDV infected colostrum deprived male Holstein calves at four different time points: prior to infection (day 0) and at 4, 9, and 16 days postinfection. In accordance with short duration of fever and lymphopenia, the expression levels of two circulating miRNAs (Bta-miR-423-5p and Bta-miR-151-3p) were different between BVDV challenged group and control group across time examined. However, only the expression level of Bta-miR-151-3p was significantly increased at the time point of 9 days postinfection compared to the control animals. Thus, these two miRNAs could not be considered as appropriate diagnostic biomarkers (58). It is widely known that persistently infected (PI) animals are the primary source of BVDV infection, and eliminating PI animals is the main measure of prevention and control of BVD. Thus, identification of different level of expression of circulating miRNAs in serums of PI cattle could be an idea approach for seeking diagnostic biomarkers of this disease.

### Ebola

The disease of Ebola caused by Ebola virus (EBOV) is a hemorrhagic infectious disease. This disease usually causes a severe hemorrhagic fever in humans and non-human primates. Early diagnosis of EBOV is of significant importance for implementation of effective interventions and prevention of the spread of infection. However, EBOV-infected patients usually do not exhibit typical symptom at the early stage for diagnosis; therefore, current diagnostic methods, such as detecting viral RNA or antigen in suspected patients, are only effective at the late stage (59).

Chen and colleagues identified a miRNA-like fragment (miR-VP-3p) generated by EBOV in the serum of Ebola patients, which was detectable before EBOV genomic RNA in the serum. Thus, this miRNA-like fragment may sever as a biomarker for early diagnosis of this disease (59). In another study, Duy and colleagues tested the circulating miRNA profiles in both rhesus macaques and humans. In total, eight circulating miRNAs (hsa-miR-146a-5p, hsa-miR-18b-5p, hsa-miR-21-3p, hsa-miR-22-3p, hsa-miR-29a-3p, hsa-miR-432-5p, hsa-miR-511-5p, and hsa-miR-596) were selected as an EBOV classifier. Further studies indicated that this classifier could correctly categorized infection status in 86% human and rhesus macaque samples (64/74). More importantly, 50% (6/12) presymptomatic rhesus macaques could also be diagnosed using this classifier (60). Using circulating miRNAs (or miRNA-like fragment), the two early diagnosis methods for Ebola will significantly promote controlling of fulminating infectious disease in the world. In addition, the finding of miR-VP-3p was a powerful evidence that RNA viruses were able to express miRNA-like small RNAs.

### Paratuberculosis

Paratuberculosis (also called Johne’s disease) is a chronic disease caused by MAP. This disease can cause substantial economic losses to cattle industry due to increased premature culling, replacement costs, decreased milk yield, reduced feed conversion efficiency, fertility problems, reduced slaughter values, and increased susceptibility to other disease or conditions. However, the lack of accurate and reliable diagnostic tests is the main challenge faced in the control of paratuberculosis in cattle (61).

To identify novel diagnostic and prognostic miRNA biomarkers, Farrell and colleagues applied RNA-seq approaches to detect the circulating miRNA profiles in serum from an experimental JD infection model (six experimental MAP-challenged calves and six age-matched controls). However, the miRNA expression levels were almost the same when comparing serum collected from MAP-challenged animals to the control group at 6 months postinfection (62). In another study, the circulating miRNA expression profiles of seropositive cattle (*n* = 5) and seronegative cattle (*n* = 7) were also detected at both early and late stages of infection, while no significant differentially expressed circulating miRNAs were detected (7). Recently, Malvisi and colleagues investigated the miRNAs related to MAP infection, and seven miRNAs (bta-mir-19b, bta-mir-19b-2, bta-mir-1271, bta-mir-100, bta-mir-301a, bta-mir-32, and Novel:14_7917) were reduced and two (bta-mir-6517 and bta-mir-7857) increased in positive animals vs. unexposed animals (63). However, it is worth noting that Malvisi and colleagues studied the miRNA profiles of whole blood, rather than circulating miRNAs of serum or plasma. As is known to all, many components in the whole blood are rich in miRNA; thus, there is no comparison between this study and the two previous ones.

### *Staphylococcus aureus* Infection

*Staphylococcus aureus* is one of the most prevalent pathogens causing chronic intramammary infection in cattle, responsible for substantial milk, quality, and economic loss in dairy farming worldwide. Sun and colleagues investigated the miRNA expression profiles of milk exosomes from four Holstein cows, whose mammary gland was infected with *S. aureus*, during mid-lactation prior to and after infection (48 h). Two miRNAs (bta-miR-142-5p and bta-miR-223) were identified as the potential indicators for early diagnosis of bacterial infection of the mammary gland (64).

### Echinococcosis

Echinococcosis, caused by *Echinococcus granulosus* sensu lato (cystic echinococcosis) or *Echinococcus multilocularis* (alveolar echinococcosis), has become a major population health problem and economic importance worldwide. About 466 million people living in Asian region, over half exposed inhabitants (in particular herdsman and farmers), are at high risk of infection with *E. granulosus* and *E. multilocularis* (65).

As antibodies of this pathogen are unable to be detected at the early stages of infection (66), effective biomarkers for early and specific diagnosis of *E. multilocularis* are urgently needed. To find potential diagnostic targets for echinococcosis, Guo and colleagues compared the circulating miRNA expression profiles in the serum collected from the *E. multilocularis*-infected and uninfected mice at 4 weeks post-infection, using RNA sequencing method. In total, the expression levels of 58 circulating miRNAs were different, of which 21 were upregulated and 37 were significantly reduced.

In addition, the expression levels of six circulating miRNAs, which were verified to be differently expressed at 4 weeks postinfection, were further analyzed at different time points postinfection. The expression levels of mmu-miR-107-3p, mmu-miR-103-3p, mmu-miR-146a-5p, and mmu-miR-21a-3p were found to be significantly increased compared with the early stage of infection. The expression level of mmu-miR-339-5p was significantly increased at the early stage of infection, while no difference was observed after 4-week infection. On the contrary, mmu-miR-222-3p was significantly down regulated in the course of *E. multilocularis* infection (67).

### Porcine Whipworm

The *porcine whipworm, Trichuris suis*, which is epidemic all over the world, lives in the large intestine of pigs. The pigs infected with this pathogen are usually subclinical, but worm loads are usually related to reduced feed efficiency, reduced growth rates, hemorrhagic diarrhea, and death (68).

To find circulating miRNAs of importance in *T. suis* infections in pigs, Hansen and colleagues investigated the expression levels of 16 preselected circulating miRNAs in serum samples collected from infected and uninfected pigs at different time points postinfection. The expression level of one circulating miRNA, ssc-let-7d-3p, was significantly increased in infected pigs at 8 weeks postinfection. As the pre-patent period of *T. suis* is 6–7 weeks, this miRNA operating at 8 weeks cannot be detected pre-patent infection with *T. suis*. Thus, its usefulness as a biomarker for early diagnosis may be limited (69).

## Circulating Parasite-Derived miRNAs

Currently, more and more miRNAs have been identified in parasitic helminths, and many of them can be detected in serum and plasma samples. Thus, these worm-derived miRNAs could be used as potential biomarkers for the early detection of particular helminth infections (70).

Guo and colleagues compared the expression levels of circulating miRNAs in the serum from the *E. multilocularis*-infected and uninfected mice, and 2 *E. multilocularis*-derived circulating miRNAs (emu-miR-10 and emu-miR-277) were identified in all 15 *E. multilocularis*-infected serum (67).

To find *schistosome*-specific miRNAs as potential biomarkers for the diagnosis of *Schistosomiasis*, Cheng and colleagues detected the miRNAs expression profiles in the plasma from rabbits infected with *Schistosoma japonicum* by a deep sequencing method. They identified five *schistosome*-specific miRNAs (Bantam, miR-3479, miR-10, miR-3096, and sja-miR-8185) in the plasma of rabbits infected with *S. japonicum*, and four of the five *schistosome*-specific miRNAs were also detected in the plasma of *S. japonicum*-infected mice. Among these *schistosome*-specific miRNAs mentioned above, miR-10 showed diagnostic potential for *S. japonicum* infection, while this result is still needed to be further confirmed in other animal models (71).

Table 1 provides summary information on circulating miRNA biomarker studies for diagnosis of veterinary infectious diseases.

**Table 1:** Circulating microRNAs (miRNAs) profiled in selected studies of veterinary infectious diseases.

Name of disease	Biological fluid	Notable miRNAs detected	Reference
Foot-and-mouth disease	Serum	bta-miR-17-5p, bta-miR-1281, bta-miR-31	Stenfeldt et al. (53)

Bovine viral diarrhea	Serum	bta-miR-423-5p, bta-miR-151-3p	Taxis et al. (58)

Ebola virus disease	Serum/plasma	miR-VP-3p, hsa-miR-146a-5p, hsa-miR-18b-5p, hsa-miR-21-3p, hsa-miR-22-3p, hsa-miR-29a-3p, hsa-miR-432-5p, hsa-miR-511-5p, hsa-miR-596	Chen et al. (59), Duy et al. (60)

*Staphylococcus aureus* infection	Milk exosomes	bta-miR-142-5p, bta-miR-223	Sun et al. (64)

Echinococcosis	Serum	mmu-miR-107-3p, mmu-miR-103-3p, mmu-miR-146a-5p, mmu-miR-21a-3p, mmu-miR-339-5p, mmu-miR-222-3p, emu-miR-10, emu-miR-277	Guo and Zheng (67)

Porcine whipworm	Serum	ssc-let-7d-3p	Hansen et al. (69)

*Schistosomiasis*	Plasma	Bantam, miR-3479, miR-10, miR-3096, and sja-miR-8185	Cheng et al. (71)

## Conclusion

In this review, recent works of circulating miRNAs involved in diagnosis of veterinary infectious diseases were introduced. Compared to thousands of published articles about utilization of circulating miRNAs as diagnostic biomarkers in human diseases, researches focusing on circulating miRNAs in animals of veterinary relevance are still deficient. This may be mainly due to that the cost of circulating miRNAs detection is much more expensive than traditional diagnostic methods in domestic animal diseases. However, the greatest advantage of circulating miRNAs is that it could be used as markers for the early detection. Upon outbreak of infection diseases, this method could quickly identify infected animals so as to prevent the spread of the epidemic and reduce economic losses, particularly for animals of high economic value, such as breeding stocks and dairy animals. In the future, with the advances in molecular biology, it is possible that the circulating miRNAs detection method will be moved from the molecular biology lab to the clinic.

In addition, in term of human disease studies, most miRNA biomarkers reported in the literature have failed to enter clinical practice because of inconsistent and irreproducible findings. One of the important reasons is that the characteristics of clinical cases (such as age, gender, ethnicity, and medical history) are usually uncontrollable. Unlike human studies, the use of experimental animals in veterinary studies can be standardized according to the needs of experimental design, which might make the veterinary related research results more repeatable and more accurate. Thus, circulating miRNAs will have a broad application prospects in the field of veterinary diseases diagnosis.

## Author Contributions

HD drafted the initial manuscript. All authors have read and approved the version to be published.

## Conflict of Interest Statement

The authors declare that the research was conducted in the absence of any commercial or financial relationships that could be construed as a potential conflict of interest.
